# Current and future opportunities of autodissemination of pyriproxyfen approach for malaria vector control in urban and rural Africa

**DOI:** 10.12688/wellcomeopenres.19131.1

**Published:** 2023-03-16

**Authors:** Augustino Thabiti Mmbaga, Dickson Wilson Lwetoijera

**Affiliations:** 1 Environmental Health and Ecological Science Department, Ifakara Health Institute, Ifakara, Morogoro, P.O. Box 53, Tanzania; 2 School of Life Sciences and Bio Engineering, Nelson Mandela African Institution of Science and Technology, Tengeru, Arusha, P.O. Box 447, Tanzania

**Keywords:** Autodissemination, Pyriproxyfen, Malaria, Larval source management

## Abstract

Despite the progress made in reducing malaria burden, new ways to address the increasing challenges of insecticide resistance and the invasion and spread of exotic malaria vectors such as *Anopheles stephensi* in Africa are urgently needed. While African countries are adopting larviciding as a complementary intervention for malaria vector control, the autodissemination technology has the potential to overcome barriers associated with the identification and treatment of prolific habitats that impede conventional larviciding approaches in rural settings. The autodissemination technology as a “lure and release” strategy works by exploiting the resting behavior of gravid mosquitoes to transfer lethal concentration of biological or chemical insecticide such as pyriproxyfen (PPF), an insect growth regulator (IGRs) to their oviposition sites and result in adult emergence.

Despite the evidence of the autodissemination approach to control other mosquito-borne diseases, there is growing and promising evidence for its use in controlling malaria vectors in Africa, which highlights the momentous research that needs to be sustained. This article reviews the evidence for efficacy of the autodissemination approach using PPF and discusses its potential as efficient and affordable complementary malaria vector control intervention in Africa. In the previous studies that were done in controlled semi-field environments, autodissemination with PPF demonstrated its potential in reducing densities of captive population of malaria vectors such as *Anopheles gambiae* and *Anopheles arabiensis. *Of importance, empirical evidence and biology-informed mathematical models to demonstrate the utility of the autodissemination approach to control wild populations of malaria vectors under field environment either alone or in combination with other tools are underway. Among others, the key determining factors for future introduction of this approach at scale is having scalable autodissemination devices, optimized PPF formulations, assess its integration/complementarity to existing conventional larviciding, and community perception and acceptance of the autodissemination approach.

## Introduction

Wide coverage and use of control interventions such as Insecticide treated bed nets (ITNs) and indoor residual spraying (IRS) have accelerated the gains in malaria burden reduction ^1, 2^. These core interventions are threatened by increasing insecticide resistance development mainly to pyrethroid class in targeted mosquito species ^3, 4^, shift to mosquitoes biting behavior to earlier hours of the evening and morning ^5– 7^, and outdoor mosquito biting when people aren’t protected ^8^.

To sustain these gains, WHO recommended larval source management (LSM) as a supplementary intervention to the core interventions ^9^. LSM, particularly larviciding, has proved to be the potential in accelerating vector density reduction in areas where mosquitoes’ breeding habitats are few, fixed, and findable ^10^. Larviciding as an ecological method can reduce densities of both endophilic and exophilic vector populations ^11^ as well as malaria incidence and prevalence in selected settings ^12, 13^. Despite these notable progress and its historical success in malaria control, its wider application in rural African settings to cover productive cryptic breeding habitats requires different innovative strategies for its deployment ^14^.

The major malaria vectors in Sub Saharan Africa are *An. gambiae, An. arabiensis, Anopheles coluzzii* and *Anopheles funestus* ^15^. Studies have shown that the majority of residual malaria transmission is mainly mediated by *An. funestus* ^16– 18^ and *An. arabiensis* ^8, 19^, which exhibit flexible behaviors including exophily ^20^ exophagy, and zoophagy ^21, 22^. Despite the efforts placed in controlling mosquitoes indoor, it is clear from the literature that ability of susceptible *An. funestus* and *An. arabiensis* to penetrate the bednets ^23^, early indoor biting ^7, 24^, outdoor biting ^25^ as well as day biting ^26^ could evade the control efforts and sustain the transmission. Moreover, while continuing to deliver interventions that tackle indoor transmission, significant attention should be focused on mosquito behavior change, including outdoor biting and early indoor biting ^7, 8^. While investments in developing outdoor-based interventions are urgently required to sustain progress made on malaria control and elimination ^27– 29^, it is also important to consider the pertinent ecological and behavioral adaptations of the dominant malaria vectors ^30^.

Promisingly, there has been an increase in designing and testing different outdoor mosquito control tools in recent years ^31^. This includes the application of insecticides to alternative hosts such as livestock ^21^; assessing the potential use of large-scale spatial repellents ^11, 32, 33^; development of outdoors odor-baited traps ^34^; attractive targeted sugar baits ^35^; topical repellents ^36^; use of genetically modified mosquitoes ^37^; eave-ribbon technology ^38^, as well as the autodissemination approach ^39^. This review discusses the opportunities and challenges of autodissemination approach with pyriproxyfen for controlling malaria vectors in urban and rural Africa.

## The autodissemination approach

Autodissemination approach is the management method that involves co-opting host seeking, ovipositing and resting adult female gravid mosquitoes’ behavior to transfer a lethal concentration of chemical insecticide such as pyriproxyfen to the breeding habitats and consequently results to adult emergence inhibition ^40, 41^.

The impetus for testing autodissemination approach for malaria vector control was inspired by Itoh and others who first showed that *Aedes aegypti* mosquitoes can be co-opted to autodisseminate insecticide ^42^. Later on, in Peru, Devine and others convincingly demonstrated a 98% larval mortality and 42–98% adult emergence inhibition, with only 4% coverage of *Aedes aegypti* resting sites with pyriproxyfen ^40^. Similar empirical benefits were also documented for Asian tiger mosquito, *Aedes albopictus* ^41, 43^. The successful demonstration of this novel mosquito assisted-larviciding approach in controlling non-malaria disease vectors has paved the way for investigating its utility for controlling malaria vectors.

## The autodissemination technology for malaria control

Large semi-field cage studies have proven the efficacy of autodissemination with pyriproxyfen approach in controlling malaria vectors; *An. gambiae* ^44^ and *An. arabiensis* ^39^. These studies demonstrated that captive population of malaria vectors can successfully pick up the pyriproxyfen particles from contamination surfaces and retain it until reaching a breeding habitat, where during the oviposition process, they contaminated the breeding habitat and render it unproductive. Using captive and stable self-sustaining populations of *An. arabiensis*, Lwetoijera and others demonstrated that only two clay pots that has been treated once with 10% active ingredient of pyriproxyfen were sufficient to crash the entire population within months of exposure ^45^. This impact, that was recorded in a relatively small surface area of approximately 184 m ^2^ covered by 800 L of water in readily available breeding habitats is likely to have been delivered by a relatively small proportion of blood fed *An. arabiensis* that prefer to rest in clay pots ^45^. Under expansive real-life settings, clay pots might compete with other nearby vegetations for resting mosquitoes. To that end, it becomes critical to ensure that purpose-built or contamination stations/devices treated with pyriproxyfen are highly attractive to visiting and resting mosquitoes, and the formulations used are efficacious and optimal to work in ultra-dosage.

There have been concerted efforts to generate highly optimized formulation of pyriproxyfen using microencapsulation technology. Previous analysis has demonstrated that microencapsulation is feasible for formulating insecticides for autodissemination and direct application into the aquatic habitats ^46, 47^. Some of the advantages offered by this innovative formulation include increased percentage of active ingredient (AI) within the formulation, thus reducing the number of mosquitoes that must be recruited to disseminate effective amounts of the formulation to the breeding habitats. In this way, microencapsulation has potential to increase formulation longevity in the field by protecting the AI from degradation by ultraviolet light, and reduce amount of insecticide and frequency of treating the autodissemination stations.

Moreover, electrostatic coating technology can be employed in the dissemination devices to increase the proportion of particles adhered to a mosquito’s body ^48^. The technology has already been used in pest control, for example in controlling sweet potato whitefly ( *Bemisia tabaci*) ^49^. In addition, electrostatic technology has been proved to increase the amount of insecticides particles taken by a single mosquito, enhancing bioavailability, and therefore a lower dose can be effective against mosquito consequently breaking resistance ^48^.

Furthermore, numerous prototypes of autodissemination devices are being developed and tested for their efficacy in controlling vector population. Performance of a novel autodissemination station/device might depend on the attractiveness of the station to mosquitoes; pick-up rate of chemical by the mosquitoes as well as dissemination of picked chemicals to targeted breeding habitats ^41^. It will also be worth considering device design with no/or minimal maintenance requirements, low cost and low risk to people who might come in contact with it ^41^. Consideration has been made with regard to the mentioned factors for a good autodissemination device; for example the development of In2Care® trap ^50^, and promising results from the assessment of its efficacy in controlling *Aedes aegypti* and *Aedes albopticus* population in both semi-field and field evaluation settings ^51– 53^. Most of the autodissemination devices have been designed and tested for mosquito species other than *Anopheles* species ^54, 55^.

With good performance in controlling *Aedes* mosquitoes, it’s important to test the effectiveness of these available autodissemination devices in controlling malaria vectors. Lwetoijera and others used the clay pots as the autodissemination devices in semi-field settings for *An. arabiensis* ^39^. Worryingly, the clay pots are fragile and impractical for large-scale deployment, but also faces competition from other resting sites in the field environment, which necessitate a new design that is more attractiveness to resting mosquitoes.

Owing to the paucity of breeding habitats during dry season, the application of autodissemination approach at this time point is likely to deliver desired impact ^56– 58^. Because mosquito abundance during dry season is low ^57, 59^, the effectiveness of autodissemination approach, items of delivering lethal dosage of pyriproxyfen to breeding habitats, is likely to be achieved *via* multiple visits of gravid mosquitoes to the breeding habitats during sequential egg laying cycles by targeted *Anopheles* specie ^60^. In addition, *Culex* mosquitoes that often share breeding habitats with Anopheles has potential to improve coverage of the target breeding habitats with pyriproxyfen ^57, 61^.

Despite of high mosquito densities associated with rainy season, application of autodissemination is likely to be impractical due to plethora and expansive nature of the breeding habitats in which the lethal concentration of pyriproxyfen cannot be achieved ^60, 62^. Failure to achieve lethal concentrations at the habitats might amplify pyrethroid resistance in mosquito population exposed to sub-lethal dosage of pyriproxyfen ^63^. Table 1 below summarizes studies on use of autodissemination with different IGR for malaria vector control.

**Table 1.** List of studies on autodissemination technology with different insect growth regulator (IGR) for controlling different malaria vectors.

SN	Study	Country	Method details	Year	Target mosquito Species	Insect growth regulator	Conclusion	References
1	Autodissemination of pyriproxyfen suppresses stable populations of *Anopheles arabiensis* under semi-controlled settings	Tanzania	Semi field experiment	2019	*An. arabiensis*	Pyriproxyfen	Suppression of stable populations of malaria vectors using a small number of simple autodissemination devices.	45
2	Effective autodissemination of pyriproxyfen to breeding sites by the exophilic malaria vector *Anopheles* * arabiensis* in semi-field settings in Tanzania	Tanzania	Semi field experiment	2014	*An. arabiensis*	Pyriproxyfen	*Anopheles arabiensis* effectively autodisseminated PPF to breeding habitats	39
3	Predicting Scenarios for Successful Autodissemination of Pyriproxyfen by Malaria Vectors from Their Resting Sites to Aquatic Habitats; Description and Simulation Analysis of a Field- Parameterizable Model	Tanzania	Mathematical Modelling	2015	Not assessed	Pyriproxyfen	Autodissemination technology can effectively eliminate malaria transmission s during the dry season with effective contamination of aquatic habitats and retain PPF activity for one week	62
4	Testing a pyriproxyfen auto-dissemination station attractive to gravid *Anopheles* * gambiae* sensu stricto for the development of a novel attract-release- and-kill strategy for malaria vector control	Kenya	Semi field experiment	2019	*Anopheles* * gambiae s.s*	Pyriproxyfen	Designed bait stations successfully attracted gravid females which were subsequently dusted with effective levels of PPF.	44

## The autodissemination technology over conventional larviciding

Conventional larviciding with biological larvicides has worked best as a complementary malaria control intervention ^58^. Implementation of the larviciding program in several sub-Saharan African countries has greatly reduced the malaria burden ^12^. For effective larviciding, WHO recommends, application to be done in areas where larval habitats are fixed, few, and findable, often found in urban settings ^9^. Hence, numerous breeding habitats that are often scattered and hard to locate have remained to be major challenges for larviciding programs in peri-urban and rural settings ^14, 64^. Even with strong political will, effective community engagement and participation, some of these operational challenges might not be full addressed. As solution options, the use of unmanned aerial vehicles (UAVs) to identify breeding habitats for larviciding at wider scales is increasingly proposed ^65– 67^. However, long processing time and technical skills required to operate UAV and handle generated data, and the need to map all the targeted breeding habitats may hamper the scale-up of this approach ^66, 68^.

Alternatively, autodissemination with pyriproxyfen has the potential to complement conventional larviciding through coverage amplification of cryptic, myriad and hard to reach breeding habitats ^69, 70^. As promising as it may sound, significant and cost-effective contribution of combining the two approaches, its impact of entomological and epidemiological disease outcomes highlights the gaps that needs to be explored.

## Pyriproxyfen as the biorational pesticides for autodissemination approach

Pyriproxyfen is a juvenile hormone analog, that interferes with the metamorphosis of mosquitoes and therefore prevents adult emergence ^71^ with an additional benefit of sterilizing female mosquitoes ^72– 74^. A miniscule PPF amount of 50 ppb has been approved for mosquito control. This amount is six times below the maximum recommended limit of 300 ppb in drinking water ^75^. Among other factors, low mammalian toxicity, safety to aquatic organisms ^76, 77^ and a long-term persistency up to 6 months in the field breeding habitats ^78^, makes the use of PPF in larviciding programs advantageous.

Pyrethroid resistance to malaria vectors has been reported to be mostly of metabolic origin ^3, 4^. Despite that PPF is being metabolized in a same way as pyrethroids ^79, 80^, and no evidence of resistance development in malaria vectors against PPF has been documented ^81^. However, there is a need to closely monitor resistance of this novel larvicide ^80^.

## Potential integration to current malaria interventions

Autodissemination (mosquito-assisted larviciding) has the potential to complement the existing frontline malaria interventions in rural and urban settings. LLINs and IRS have contributed nearly 40% of the 57% reduction in incidence of clinical diseases, representing over 81% relative contribute to the success in malaria control for the past two decades ^1^ and 23% reduction in child mortality across most endemic sub-Saharan countries ^82^. These assuring progress has further fueled the development of next generation LLINs and chemistries for IRS to ensure its usefulness against resistant mosquitoes populations ^83– 85^.

The use of autodissemination with pyriproxyfen has potential to complement these tools by controlling mosquitoes at its breeding habitats. Convincingly, the use of pyriproxyfen *via* conventional larviciding has already demonstrated effectiveness in preventing emergence of *An. arabiensis* and *An. funestus* at experimental scale in the field settings ^86, 87^. Pyriproxyfen can also sterilize adult malaria vectors through contact with treated nets, such as Olyset Duo nets ^88– 90^. Of importance, it has been demonstrated that sterilized mosquitoes can still transfer PPF sufficient to prevent adult emergence at the contaminated habitats (H. Kunambi, personal communication). This development, highlight the unique complementarity of autodissemination with bednets co-treated with pyriproxyfen.

Habitat-based modelling by Gu and Novak highlighted that in order to combat malaria in Africa, larval interventions should be focused in identifying and targeting prolific habitats ^91^, a task that might be accomplished with nearly perfection using autodissemination approach ^69, 70^. Hence, it is our expectations that eventual deployment of autodissemination with pyriproxyfen might further accelerate these control efforts by amplifying the coverage of pyriproxyfen to the productive breeding habitats. While studies to demonstrate the entomological impact of autodissemination with pyriproxyfen under field settings where LLINs are widely used are ongoing, future trials should also aim to establish the combined impact of autodissemination and LLINs and/or other interventions to understand whether the overall effect would be synergistic, additive, or antagonistic.

Autodissemination technology itself as a novel vector control tool, can be integrated into other malaria intervention in a cost-effective way. Autodissemination with PPF can contribute to tools box for controlling *Anopheles stephensi* in urban settings. Since its first report in Djibouti ^92^, *An. stephensi* has spread to Ethiopia, Somalia and Sudan and Nigeria ^2^ and more recently in Kenya (E. Ochomo, unpublished report). Similar to *Aedes* mosquitoes, *An. stephensi* mostly breed in man-made water storage containers and discarded wastes ^93^. Successful establishment of this species in urban settings, where discarded wastes are ubiquitous and poorly managed, will pose great challenge to malaria control efforts. The geo-statistical model predicted the “worst case scenario” where by more than 126 million people residing in African cities will be at risk of contracting malaria if no action is taken ^94^. Scientists have argued that; instead of addressing the threat as stand alone, it’s important to use integrated response that can also target other malaria vectors, and hence proper utilization of resources to fit different contexts ^95^. Similar to the successes made in controlling *Aedes* mosquitoes using autodissemination with pyriproxyfen elsewhere ^96^, this approach is well suited and can be effectively integrated into conventional larviciding programs to specifically target breeding habitats of *An. stephensi*. This approach might be more applicable and cost-effective in urban settings with low transmission and where widespread of LLINs is unjustified ^97^.

Impact of community involvement in malaria control should never be underrated. Malaria control needs strong collaboration among different expertise including the target communities ^98^. The sensitive role of the community in mosquito control is well documented ^99, 100^. Similar to other malaria interventions, scaling up autodissemination technology shall strongly need community participation, to get the desired impact. However, the evidence of engaging communities with regards to PPF-based studies is limited and therefore needs to be explored ^101^.

Because the autodissemination stations will be placed outside near human dwellings, raising awareness to the community on the safety of PPF to humans especially children that might come into contact with the stations is critical. Contamination of the environments, including human and animal drinking water with PPF deposited by contaminated mosquitoes, will also necessitate community involvement, approval and ownership of the autodissemination approach in their locality.

The autodissemination technology will be deployed in parallel with environmental cleaning that will not only reduce vegetations exploited by resting mosquitoes, but also maximize mosquito resting time in the provided autodissemination stations. For this reason, it will be important to encourage and empower communities with trainings on household environmental cleaning that will directly reduce mosquito densities and associated bites ^102^.

## Conclusion

Evidences supporting the future use of autodissemination approach with pyriproxyfen for malaria control are increasingly documented but more studies on field validation of this approach to formulate its target product profile are required. Among factors that should be looked at include scalable autodissemination devices with potential to target wide range of *Anopheles* behavior. Moreover, effective PPF formulations that not only can be easily picked and off-loaded with mosquitoes, but also permit extended persistence at different application surfaces of autodissemination devices and in different water quality (polluted/unpolluted) of breeding habitats is critical for the success of autodissemination approach.

### Ethical approval

The Institutional Review Board of the Ifakara Health Institute (IHI/IRB/EXT/No: 17-2022) and the Medical Research Coordination Committee of the National Institute for Medical Research in Tanzania (NIMR/HQ/R.8c/Vol.I /2235) approved the study.

## Data Availability

No data are associated with this article. DWL and ATM conceptualized the idea, curated the reviewed articles, wrote the original review and approved the final review version. Augustino Thabiti Mmbaga: *Conceptualization, Data Curation, Writing – Original Draft Preparation* Dickson Wilson Lwetoijera: *Conceptualization, Data Curation, Writing – Original Draft Preparation*
