# Supplementary material for: Current and future opportunities of autodissemination of pyriproxyfen approach for malaria vector control in urban and rural Africa
Source: Wellcome Open Res. 2023 Oct 30;8:119. Originally published 2023 Mar 16. [Version 3] doi: 10.12688/wellcomeopenres.19131.3 (PMC10333782; doi:10.12688/wellcomeopenres.19131.3)
Supplement: Peer review history

# Reviewer report: Reviewer response for version 2

**DOI:** 10.21956/wellcomeopenres.21478.r61190

**Published:** 2023-07-10

**Authors:** Rina Tilak

**Affiliations:** 1 Armed Forces Medical College, Pune, Maharastra, India

The article is relevant and important in the current context of malaria elimination goals. A well-written article. There are two technical queries and a few typographical suggestions which have been highlighted. 

**Technical: **

- The authors on page 6 para 2 have mentioned that larviciding with PPF and adult control through PPF-treated LLIN will complement each other. Will exposure at both stages not enhance the development of resistance especially given the fact that pyrethroid resistance will be affected? Please clarify. 
- Para 2 on the same page, the authors have commented that larval interventions should be focussed...a task that might be accomplished using autodissemination, please clarify how this will be achieved?
- Conclusion - line 5 - Among factors that should be looked at include scalable autodissemination devices...to target wide range of Anopheles behaviour - can the authors explain which behaviour they are referring to?

**Typographical:**

 The article is well written, however, a few minor typographical corrections are needed. which are as under:

1. Abstract: sentence three needs to be amended with the addition of word inhibition - The autodissemination technology as a lure and release...adult emergence [inhibition].
2. Page 3, Introduction: subheading- "The autodissemination approach" - Please italicize *Aedes albopictus*. Please add ref to the statement that "Limited evidence suggests that adult *Aedes albopictus* males could deliver..."
3. Page 3, Introduction subheading- "The autodissemination technology for malaria control" - Please amend the sentence, "These studies demonstrated...particles from contaminated..., they contaminate..."
4. Page 4, para 4- last line design that is "more attractive" instead of "attractiveness".
5. Page 4 second column first para - line 7 - " *Anopheles *species" (instead of "specie"); line 8 - " *Anopheles *have" instead of "has"
6. Page 4 second column second para - line 1 delete "of" after despite; delete "below" after Table 1.
7. Page 4 second column third para - line 9 after "remained" delete "to be" and "add as". 
8. Page 4 second column fourth para - line 5 "impact of" should be - "impact on".
9. Page 5 second column - line 5 instead of "contribute" it should be "contribution".
10. Page 6 para 2 - line 12 - it should be "highlights" instead of "highlight"; para 3 line 4 - it should be "near perfection" and not "nearly"; line 5 it should be "expectation" and not "expectations"; para 4 line 2 amend can be integrated with other instead of into other; same para line 16 - remove colon after "scientists have argued that".
11. Page 6 second column first para - last line - add "use after widespread"; second para rephrase second sentence to make it more clear - "Malaria control needs..."

Is the review written in accessible language?

Yes

Are all factual statements correct and adequately supported by citations?

Yes

Are the conclusions drawn appropriate in the context of the current research literature?

Yes

Is the topic of the review discussed comprehensively in the context of the current literature?

Yes

Reviewer Expertise:

Vector borne diseases

I confirm that I have read this submission and believe that I have an appropriate level of expertise to confirm that it is of an acceptable scientific standard.

# Response

**Published:** 2023-10-13

**Authors:** Dickson Wilson Lwetoijera

**Affiliations:** Ifakara Health Institute, Tanzania

We thank the reviewer for their thoughtful and constructive comments on this manuscript. In the revised version, we have made the following changes and possible clarifications on technical queries and typographical suggestions as per the reviewers’ comments.

**Technical**

**Comment 1**: The authors on page 6 para 2 have mentioned that larviciding with PPF and adult control through PPF-treated LLIN will complement each other. Will exposure at both stages not enhance the development of resistance especially given the fact that pyrethroid resistance will be affected? Please clarify.

**Response**: At the population level, mosquitoes will be sterilized following exposure to LLINs treated with PPF and will be inhibited from emerging in breeding habitats. To date, there is limited evidence on the effect of PPF on pyrethroid resistance; a previous study exploring the effect of sub-lethal PPF doses on pyrethroid resistance in *Anopheles arabiensis* was only performed for a single generation, which makes it unclear whether the observable magnification of pyrethroid resistance is just tolerance or actual resistance that can be inherited over multiple generations.

**Comment 2**: Tilak: Para 2 on the same page, the authors have commented that larval interventions should be focussed a task that might be accomplished using autodissemination, please clarify how this will be achieved?.

**Response:** A task that might be accomplished with near perfection using autodissemination approach, based on the evidence that gravid mosquitoes know better their most preferred habitats

**Comment 3**: Conclusion - line 5 - Among factors that should be looked at include scalable autodissemination devices...to target wide range of Anopheles behaviour - can the authors explain which behaviour they are referring to?

**Response**: In the line 5 of conclusion section, the Anopheles behaviors that the authors referred to were mating, host-seeking, resting, and oviposition.

**Typographical**

**Comment 1**: Abstract: sentence three needs to be amended with the addition of word inhibition - The autodissemination technology as a lure and release...adult emergence [inhibition].

**Response**: The word “inhibition” has been added in sentence three of the abstract, which now reads “The autodissemination technology as a “lure and release” strategy works by exploiting the resting behavior of gravid mosquitoes to transfer lethal concentration of biological or chemical insecticide such as pyriproxyfen (PPF), an insect growth regulator (IGRs) to their oviposition sites and result in adult emergence inhibition”.

**Comment 2**: Page 3, Introduction: subheading- "The autodissemination approach" - Please italicize Aedes albopictus. Please add ref to the statement that "Limited evidence suggests that adult Aedes albopictus males could deliver..."

**Response:** The Species name *Aedes albopictus* has been italicized. Also, the article by Mains et al., 2015 has been added as a reference for the sentence that reads “Limited evidence suggests that adult *Aedes albopictus* male could directly deliver PPF to the breeding habitats or cross contaminate the females that will eventually deliver it to breeding habitat (Mains et al., 2015).

**Comment 3**: Page 3, Introduction subheading- "The autodissemination technology for malaria control" - Please amend the sentence, "These studies demonstrated...particles from contaminated..., they contaminate...".

**Response:** The word “contamination” has been replaced with “contaminated” and “contaminated” replaced with “contaminate” in the introduction subheading section, page 3.

**Comment 4**: Page 4, para 4- last line design that is "more attractive" instead of "attractiveness". 

**Response**: On page 4, para 4 – last line, the word “attractiveness” has been replaced with “attractive”. Now the sentence reads “Worryingly, the clay pots are fragile and impractical for large-scale deployment but also face competition from other resting sites in the field environment, which necessitate a new design that is more attractive to resting mosquitoes.

**Comment 5**: Page 4 second column first para - line 7 - "Anopheles species" (instead of "specie"); line 8 - "Anopheles have" instead of "has".

**Response**: The word “specie” has been replaced with “species”. Also, the word “has” has been replaced with “have” in page 4 second column fist para – line 7 as suggested by the reviewer.

**Comment 6**: Page 4 second column second para - line 1 delete "of" after despite; delete "below" after Table 1.

**Response**: On page 4 second column second para - line 1,  the words “of” and “below” have been deleted.

**Comment 7**: Page 4 second column third para - line 9 after "remained" delete "to be" and "add as".

**Response**: On page 4 second column third para - line 9, the words “to be” have been replaced with “as”

**Comment 8**: Page 4 second column fourth para - line 5 "impact of" should be - "impact on".

**Response: **On Page 4 second column fourth para - line 5, the word “on” has replaced “of”

**Comment 9**: Page 5 second column - line 5 instead of "contribute" it should be "contribution".

**Response:** On Page 5 second column - line 5, the word “contribution” has replaced “contribute”

**Comment 10**: Page 6 para 2 - line 12 - it should be "highlights" instead of "highlight"; para 3 line 4 - it should be "near perfection" and not "nearly"; line 5 it should be "expectation" and not "expectations"; para 4 line 2 amend can be integrated with other instead of into other; same para line 16 - remove colon after "scientists have argued that".

**Response:** In the Page 6 para 2 - line 12, the word “highlights” has been added to replace “highlight”; para 3 line 4 the word “nearly” has been replaced with “near”; on line 5 the word “expectations” has been replaced by “expectation”; also, on the para 4 line 2 the colon has been removed as suggested.

**Comment 11**: Page 6 second column first para - last line - add "use after widespread"; second para rephrase second sentence to make it more clear - "Malaria control needs..."

**Response:** The sentence has been paraphrased to read “Strong collaboration amongst different experts, including affected communities, is necessary for effective malaria control” instead of “Malaria control needs strong collaboration among different expertise including the target communities”

# Reviewer report: Reviewer response for version 2

**DOI:** 10.21956/wellcomeopenres.21478.r61194

**Published:** 2023-07-10

**Authors:** Daibin Zhong

**Affiliations:** 1 University of California at Irvine, Irvine, California, USA

The review by Mmbaga and Lwetoijera offers a thorough and impactful overview of the benefits, drawbacks, present and future potential of the autodissemination of pyriproxyfen approach for malaria vector control in both urban and rural regions of Africa. This manuscript is captivating, well-organized, and exemplifies excellent writing with properly referenced statements and a compelling conclusion. The use of autodissemination methods employing insect growth regulators (IGRs) has gained significant interest, demonstrating efficacy in controlling *Aedes *mosquitoes in various field and semi-field settings. However, limited information exists regarding its effectiveness against Anopheline mosquitoes. This review presents updated information on the autodissemination of pyriproxyfen approach for malaria vector control in urban and rural Africa. The authors emphasize the importance of key factors such as the development of scalable autodissemination devices, optimized PPF formulations, assessment of integration with existing conventional larviciding methods, and community perception and acceptance of the autodissemination approach for successful implementation at a larger scale. The manuscript is well written, statements are referenced as required and appropriate conclusion provided.

 Minor comments:

1. Please consider italicizing the genus and species names: "Anopheles" on Page 4, line 8, and References 6, 7, and 39; "Aedes" on Page 3, line 16, and Reference 43.
2. Additionally, there is a study by Okazawa *et al.* (1991) ^1^ that demonstrates the efficacy of PPF in inhibiting adult emergence of *Anopheles punctulatus*.
3. It would be worthwhile to investigate the presence of evidence supporting the effectiveness of PPF in controlling insecticide-resistant mosquitoes.
4. Considering the reported ineffectiveness of PPF for *An. quadrimaculatus* (Swale *et al. *2018), it would be valuable to explore alternative IGRs as potential options for controlling insecticide-resistant mosquitoes.

Is the review written in accessible language?

Yes

Are all factual statements correct and adequately supported by citations?

Yes

Are the conclusions drawn appropriate in the context of the current research literature?

Yes

Is the topic of the review discussed comprehensively in the context of the current literature?

Yes

Reviewer Expertise:

Vector ecology, malaria epidemiology, molecular biology, and population genetics

I confirm that I have read this submission and believe that I have an appropriate level of expertise to confirm that it is of an acceptable scientific standard.

## References

[1] : Field evaluation of an insect growth regulator, pyriproxyfen, against Anopheles punctulatus on north Guadalcanal, Solomon Islands. *J Am Mosq Control Assoc* .1991;7(4) :604-7 1787406

[2] : Development of an autodissemination strategy for the deployment of novel control agents targeting the common malaria mosquito, Anopheles quadrimaculatus say (Diptera: Culicidae). *PLoS Negl Trop Dis* .2018;12(4) : 10.1371/journal.pntd.0006259 e0006259 10.1371/journal.pntd.0006259 29641515PMC5894962

# Response

**Published:** 2023-10-13

**Authors:** Dickson Wilson Lwetoijera

**Affiliations:** Ifakara Health Institute, Tanzania

We thank the reviewers for their thoughtful and constructive comments on this manuscript. In the revised version, we have made the following changes and possible clarifications on technical queries and typographical suggestions as per the reviewers’ comments.

**Comment 1**: Please consider italicizing the genus and species names: "Anopheles" on Page 4, line 8, and References 6, 7, and 39; "Aedes" on Page 3, line 16, and Reference 43.

**Response: **The recommended changes has been made in the revised manuscript.

**Comment 2**: Additionally, there is a study by Okazawa et al. (1991)1 that demonstrates the efficacy of PPF in inhibiting adult emergence of *Anopheles punctulatus*.

**Response:** We acknowledge the reviewer for the suggestion to add a study by Okazawa et al. (1991) that demonstrated the efficacy of PPF in inhibiting adult emergence of *Anopheles punctulatus*. However, we did not include the study because it did not involve an autodissemination approach, contrary to the central theme of our review work.

**Comment 3**: It would be worthwhile to investigate the presence of evidence supporting the effectiveness of PPF in controlling insecticide-resistant mosquitoes.

**Response**: Evidence supporting the effectiveness of PPF in controlling insecticide-resistant mosquitoes has been discussed and two additional citation have been added. In page 8, sub heading titled potential integration to current malaria intervention, the reference for the sentence “Of importance, it has been demonstrated that sterilized mosquitoes can still transfer PPF sufficient to prevent adult emergence at the contaminated habitats” has been added. H. Kunambi personal communication has been replaced with the actual citation (Kunambi et al., 2023).

**Comment 4:**  Considering the reported ineffectiveness of PPF for *An. quadrimaculatus* (Swale et al. 2018), it would be valuable to explore alternative IGRs as potential options for controlling insecticide-resistant mosquitoes.

**Response:** A semi field-study by Swale and others demonstrated that *An. quadrimaculatus* can deliver a sufficient novaluron, a chitin synthesis inhibitor, to its breeding habitats via autodissemination approach and cause adult emergence inhibition (Swale et al., 2018). Until recent, major malaria vectors *An. arabiensis, An. gambiae* and *An. funestus* under semi-field settings have proven to be susceptible to novaluron (Ngonzi et al., 2022). Furthermore, it is extremely important that future research should look at the possibility to other explore alternative IGRs that could be used for autodissemination approach in rotation with pyriproxyfen.

# Reviewer report: Reviewer response for version 2

**DOI:** 10.21956/wellcomeopenres.21478.r56394

**Published:** 2023-04-21

**Authors:** Oscar Mbare

**Affiliations:** 1 International Centre of Insect Physiology and Ecology, Nairobi, Kenya

Accept

Is the review written in accessible language?

Yes

Are all factual statements correct and adequately supported by citations?

Yes

Are the conclusions drawn appropriate in the context of the current research literature?

Yes

Is the topic of the review discussed comprehensively in the context of the current literature?

Yes

Reviewer Expertise:

Malaria ecology, vector biology and vector control

I confirm that I have read this submission and believe that I have an appropriate level of expertise to confirm that it is of an acceptable scientific standard.

# Reviewer report: Reviewer response for version 1

**DOI:** 10.21956/wellcomeopenres.21208.r55603

**Published:** 2023-04-03

**Authors:** Oscar Mbare

**Affiliations:** 1 International Centre of Insect Physiology and Ecology, Nairobi, Kenya

This manuscript provides an overview of the current evidence of the potential role of auto-dissemination for malaria vector control including experiments conducted in the laboratory and large semi-field systems. Importantly, the authors have identified the factors that need to be addressed for successful implementation of the auto-dissemination strategy for control of malaria vectors. The manuscript identifies the need to conduct additional studies to increase the evidence on possible role of auto-dissemination strategy as an effective complementary tool for malaria vector control. The manuscript is well written, statements are referenced as required and appropriate conclusion provided.

 Minor issues

1. Page 3 (the sub-title “The autodissemination approach”) - Can male *Anopheles* also be targeted for transfer of pyriproxyfen? Probably discuss this in this section of the manuscript. See the article Mains *et al.,* (2015) ^1^.
2. Page 4 (the sub-title “The autodissemination technology over conventional larviciding”) – Replace the word ‘full’ with ‘fully’.
3. Add the article by “Swale et al., 2018 Development of an autodissemination strategy for the deployment of novel control agents targeting the common malaria mosquito, *Anopheles quadrimaculatus* say (Diptera: Culicidae)” ^2^ to Table 1 on the list of studies on autodissemination technology with different insect growth regulators (IGR) for controlling different malaria vectors.
4. Page 4 - Is the difficulty to identify/develop more attractive oviposition-based traps/dissemination stations another limitation in the use of auto-dissemination approach in the control of malaria vectors?

Is the review written in accessible language?

Yes

Are all factual statements correct and adequately supported by citations?

Yes

Are the conclusions drawn appropriate in the context of the current research literature?

Yes

Is the topic of the review discussed comprehensively in the context of the current literature?

Yes

Reviewer Expertise:

Malaria ecology, vector biology and vector control

I confirm that I have read this submission and believe that I have an appropriate level of expertise to confirm that it is of an acceptable scientific standard.

## References

[1] : Male mosquitoes as vehicles for insecticide. *PLoS Negl Trop Dis* .2015;9(1) : 10.1371/journal.pntd.0003406 e0003406 10.1371/journal.pntd.0003406 25590626PMC4322094

[2] : Development of an autodissemination strategy for the deployment of novel control agents targeting the common malaria mosquito, Anopheles quadrimaculatus say (Diptera: Culicidae). *PLoS Negl Trop Dis* .2018;12(4) : 10.1371/journal.pntd.0006259 e0006259 10.1371/journal.pntd.0006259 29641515PMC5894962

# Response

**Published:** 2023-04-12

**Authors:** Dickson Wilson Lwetoijera

**Affiliations:** Ifakara Health Institute, Tanzania

We do thank a reviewer for comments and construction discussion points, and here are point-by-point responses

**Comment 1:** Page 3 (the sub-title “The autodissemination approach”) - Can male *Anopheles* also be targeted for transfer of pyriproxyfen? Probably discuss this in this section of the manuscript. See the article Mains *et al.,* (2015) ^1^ .

**Response:** In the revised version we have discussed this point, but also acknowledged its practical limitations, by providing a text "Limited evidence suggests that adult *Aedes albopictus* male could directly deliver PPF to the breeding habitats or cross contaminate the females that will eventually deliver it to breeding habitat. However, this strategy is likely to be hampered with mass rearing and release of male mosquitoes" 

**Comment 2: **Page 4 (the sub-title “The autodissemination technology over conventional larviciding”) – Replace the word ‘full’ with ‘fully’.

**Response: **Revision has been made in the revised version of the article.

**Comment 3:** Add the article by “Swale *et al.,* 2018 Development of an autodissemination strategy for the deployment of novel control agents targeting the common malaria mosquito, *Anopheles quadrimaculatus* say (Diptera: Culicidae)” ^2^ to Table 1 on the list of studies on autodissemination technology with different insect growth regulators (IGR) for controlling different malaria vectors.

**Response:** Thank you for this key observation, in the revised article, we have added the suggested article Swale et al 2018 in Table 1.

**Comment 4: **Page 4 - Is the difficulty to identify/develop more attractive oviposition-based traps/dissemination stations another limitation in the use of auto-dissemination approach in the control of malaria vectors?

**Response:** We thank the reviewer for this discussion point, while we acknowledge this to be a challenge, we are also convinced that with appropriate funding and time, that highly attractive device that can effectively contaminate malaria vectors can be designed, as it has been for *Aedes *mosquitoes, and this has been discussed in the article. No changes have been made in the revised version
